# Cytotoxic, Antioxidant and Antimicrobial Activities and Phenolic Contents of Eleven *Salvia* Species from Iran 

**Published:** 2013

**Authors:** Omidreza Firuzi, Ramin Miri, Mojtaba Asadollahi, Saba Eslami, Amir Reza Jassbi

**Affiliations:** Medicinal and Natural Products Chemistry Research Center, Shiraz University of Medical Sciences, Shiraz, Iran.

**Keywords:** *Salvia*, Phenolics, DPPH, Radical-scavenging, Cytotoxic activity, Antibacterial activity

## Abstract

The plants of the genus *Salvia *synthesize several types of secondary metabolites with antimicrobial, cytotoxic, and radical scavenging activities and are used in the folk medicine of different countries. Eleven *Salvia *species including *S. aegyptiaca*, *S. aethiopis*, *S. atropatana, S. eremophila*, *S. hypoleuca*, *S. limbata, S. nemorosa, S. santolinifolia*, *S. sclarea*, *S. syriaca, *and *S. xanthocheila *were collected from different localities in Iran and screened for their cytotoxic activity using 3-(4,5-dimethylthiazol-2-yl)-2,5-diphenyltetrazolium bromide (MTT) colorimetric assay. The antioxidant potential and total phenol contents of the plant extracts were assessed by 2,2-diphenyl-1-picrylhydrazyl (DPPH) radical scavenging assay and Folin- Ciocalteu reagent respectively and finally antimicrobial activity of the above extracts were determined by using agar disc diffusion (ADD) and nutrient broth micro-dilution (NBMD) bioassays. Cytotoxic activity of methanol, 80% methanol and dichloromethane extracts of these plants were assessed on 3 human cancer cell lines. All of the extracts of *S. eremophila *and *S. santolinifolia *were active at IC_50 _values of 10.5-75.2 μg extract/mL, while the methanol and dichloromethane extracts of *S. limbata*, *S. hypoleuca *and *S. aethiopis *showed considerable cytotoxic activity against the tested cell lines. Among the tested plants for their antioxidant activity, *S. nemorosa*, *S. atropatana*, *S. santolinifolia*, and *S. eremophila *were the most active radical scavengers with higher total phenol contents while, *S. limbata*, *S. xanthocheila *and *S. aegyptiaca *were the weakest ones. The methanol extracts of *S. santolinifolia*, *S. eremophila, S. sclarea *and *S. limbata *inhibited the growth of all tested bacterial strains.

## Introduction

Sage plants (of genus *Salvia*) are known for their uses in the folk medicine and as additives in food products in different countries including Iran and Turkey (1, 2). They are widely spread in both countries and are rich in volatiles such as mono-and sesquiterpenoids (3) in their essential oil and non-volatile terpenoids especially di- and triterpenoids (4, 5). These plants also synthesize polyphenols, including flavonoids and caffeic acid derivatives (6, 7). 

The diterpenoids such as abietane pigments (5), pimarane (1) and labdane type diterpenoids, and triterpenoids together with volatile mono-and sesquiterpenoids found in their essential oils are responsible for different biological activities including antimicrobial (1, 8), cytotoxic (9, 10), enzyme inhibitory (11-13) and anti-leishmania properties (14). Some of the species of *Salvia *including *S. hypoleuca *(15) and *S. syriaca *(16) are unique sources of the rare sesterterpenoids in the terrestrial plants. The interesting biological activities resulting from their chemical diversities prompted different researchers worldwide to examine these plants for their constituents and biological activities. 

As a starting point for selection of target plants for the isolation of bioactive natural products, we subjected eleven sage species collected from different localities in Iran to antioxidant, antimicrobial and cytotoxic bioassays against human cancer cell lines. They are *S. aegyptiaca*, *S. aethiopis*, *S. atropatana, S. eremophila*, *S. hypoleuca*, *S. limbata, S. nemorosa, S. santolinifolia*, *S. sclarea*, *S. syriaca, *and *S. xanthocheila.*

## Experimental

*Reagents*

Quercetin was obtained from Acros Organics (Geel, Belgium). Fetal bovine serum (FBS), RPMI 1640, trypsin and phosphate buffered saline (PBS) were purchased from Biosera (Ringmer, UK). Dimethylsulfoxide (DMSO), Folin-Ciocalteu reagent, nutrient broth, hexane, methanol and sodium carbonate were purchased from Merck (Darmstadt, Germany). 3-(4,5-dimethylthiazol-2-yl)-2,5-diphenyltetrazolium bromide (MTT), 2,2-diphenyl-1-picrylhydrazyl (DPPH), chloramphenicol, and hydrochloric acid 32% were obtained from Sigma-Aldrich (St. Louis, MO, USA). Doxorubicin and penicillin/streptomycin were purchased from EBEWE Pharma (Unterach, Netherlands) and Invitrogen (San Diego, CA, USA), respectively. *p*-iodonitrotetrazolium violet (INT) was obtained from Fluka.

*Plant material*

Plants studied in this report were collected in June and July 2008 from different areas of Iran (Table 1) and identified at the Medicinal and Natural Products Chemistry Research Center (MNCRC), Shiraz, Iran by Dr. Mojtaba Asadollahi. The voucher specimens were deposited at MNCRC herbarium. Aerial parts of the plants were air-dried at room temperature in the shade and were used for solvent extraction. 

**Table 1:** Location and herbarium specimens of the plants species

*Plant Name *	Location	Herbarium Number	Date
*Salvia aegyptiaca *L.	Darab towards Rostagh, Fars N 28º, 35’ a E 54º, 47’ 1260 m	PC-87-90	June 2008
*Salvia aethiopis *L.	Arasbaran Forest- East Azarbaijan N 38º, 53’ E 46º, 50’ 1800 m	PC-87-91	August 2008
*Salvia atropatana *Bunge.	Cheleghah, Sepidan, Fars N 30º, 17’ E 51º, 56’ 2370 m	PC-88-19	July 2008
*Salvia eremophila *Boiss.	Darab, Fars N 28º, 41’ E 54º, 37’ 1170 m	PC-87-92	June 2008
*Salvia hypoleuca *Benth.	Kandovan-Chalus road- Mazandaran N 36º, 10’ E 51º, 18’ 2500 m	PC-88-18	July 2008
*Salvia limbata *C. A. Mey.	Shahin dej, west Azarbayjan N 36º, 39’ E 46º, 32’ 1500 m	PC-87-93	August 2008
*Salvia nemorosa *L.	Marzanabad, Chalus, Mazandaran N 36º, 27’ E 51º, 18’ 480 m	PC-88-20	July 2008
*Salvia santolinifolia *Boiss.	Darab, Fars N 28º, 41’ E 54º, 37’ 1170 m	PC-87-98	June 2008
*Salvia sclarea *L.	Sepidan (Ardakan) towards Komehr, Fars N 30º, 24’ E 51º, 54’ 2600 m	PC-87-99	July 2008
*Salvia syriaca *L.	Shiraz-Sepidan road, after shool village, Fars N 29º, 58’ E 52º, 10’ 2090 m	PC-87-100	June 2008
*Salvia xanthocheila *Boiss. ex Benth.	Kandovan-Chalus road- Mazandaran N 36º, 10’ E 51º, 18’ 2500 m	PC-87-101	July 2008

a) The approximate collection coordinates of the plants

*Solvent extraction of the plants*

The aerial part of each plant was separately extracted with dichloromethane, methanol and 80% methanol for the cytotoxic and antibacterial bioassays. Extracts were prepared as follows; 3 g of the dry plant was macerated in 60 mL of the solvents for 24 h. The extraction was repeated twice and the resulting extracts were added to each other. The extract was then filtered and concentrated in a rotary evaporator under reduced pressure for the removal of solvents. The resulting concentrated extracts were kept at -20 °C until their use for antimicrobial and cytotoxic tests. Shortly before each experiment, the syrup was dissolved in the appropriate solvent (DMSO) and used in the bioassay. The extracts that were used for antioxidant and total phenols measurements were prepared in a different way. Twenty-five mg of dried powdered plant material was extracted in 1.5 mL 80% methanol for 48 h and an aliquot of the extract without further concentration was subjected to the above-mentioned assays. 

*Cell lines and culture*

The following human cancer cell lines were purchased from the National Cell Bank of Iran, Pasteur Institute, Tehran, Iran; HL60 (human acute promyelocytic leukemia cells), K562 (human chronic myelogenous leukemia cells) and MCF-7 (human breast adenocarcinoma cells).

The cells were cultured in sterile T25 flasks in RPMI 1640 medium supplemented with fetal bovine serum (20% v/v for HL60 and 10% v/v for K562 and MCF-7 cells), penicillin (100 units/mL) and streptomycin (100 μg/mL). HL60 and K562 cell lines were grown in suspension, while MCF-7 cells were grown in mono layer cultures in humidified air constituting 5% CO_2_ at 37 °C.

*Cytotoxicity assay*

The inhibitory effect of plant extracts on cell growth was assessed by 3-(4,5-dimethylthiazol-2-yl)-2,5-diphenyltetrazolium bromide (MTT) assay. This colorimetric assay is based on the conversion of the yellow tetrazolium bromide (MTT) to the purple formazan by the action of mitochondrial enzyme succinate dehydrogenase in viable cells. The powdered extracts were dissolved in DMSO, and then diluted in growth medium at least 200 times. Cells were seeded in 96-well plates at the density of 50,000 cells/mL in 100 μL medium and incubated for 24 h. Then, 50 μL of medium was replaced with fresh medium containing 3 different concentrations of the extracts. After 72 h of incubation, the medium of each well was replaced by RPMI without phenol red containing 0.5 mg/mL MTT and incubated at 37 °C for 4 h. DMSO was used to dissolve the formed formazan crystals. The potency of cell growth inhibition for each extract was expressed as IC_50_ value, defined as the concentration that caused a 50% of maximum inhibition of cell viability. The absorbance of different wells was measured at 570 nm, with background correction at 655 nm using a microplate reader. Inhibition percentages were plotted against different concentrations of the extracts and cisplatin. The IC_50_s were calculated by best fit equations using Curve Expert statistical program.

*Determination of the free radical scavenging activity of the plant extracts by spectrophotometric methods*

The free radical scavenging activity of the plant extracts was measured by the method of Blois (17) with some modifications (18) and compared to that for quercetin as a standard radical scavenger. Briefly, 25 mg of dried powdered plant was extracted in 1.5 mL 80% methanol for 48 h. 25-100 μL of this extract were adjusted to 200 μL by methanol to obtain different concentrations of the plant᾽s extract and then added to 3800 μL 10^-4^ M DPPH solutions in methanol. After 30 min shaking of the solutions in the dark, the absorptions of the DPPH solutions were measured at 517 nm. The percentage of the reduced DPPH was calculated by the following equation:

Percentage of DPPH reduction = ((A_0_ – A_1_) / A_0_) x 100), where A_0_ is the absorbance of the control, and A_1_ is the absorbance in the presence of sample. The IC_50_s were calculated by linear regression equations of the DPPH inhibition percentage from different concentrations of the extracts and the standard antioxidants, using Microsoft Excel and Curve Expert statistical programs and expressed as μg plant material extracted with the solvent/ 1 mL 10^-4^ M DPPH (μg PM /ml DPPH). 

*Determination of the total phenol content in the plant extracts*

The total phenol contents of the plant extracts were determined by the Folin-Ciocalteu method as described previously with some modifications (19). Briefly, 3.16 mL water was added to a 40 μL solution of the plant extract (80% methanol) and 200 μL Folin-Ciocalteu reagent, and the mixture was shaken until it became homogenous. To this solution was added 600 μL of a 0.25% sodium carbonate after 8.5 min incubation at room temperature. The above solution was further incubated at RT for 2 h and its absorbance was measured at 765 nm against the blank. The concentrations of the total phenolics were measured against a series of gallic acid standard solutions and expressed as mg equivalent of gallic acid in 1g dry plant material (mg EG/g PM) (19).

*Antibacterial agar disc diffusion method*

To examine the antibacterial activity of the plant extracts, three Gram-negative bacteria (*Escherichia coli*: PTCC1330*, Klebsiella pneumonia*e: PTCC1053 and *Salmonella typhi*: PTCC1609) and three Gram-positive bacteria (*Staphylococcus aureus*: PTCC1112, *Staphylococcus epidermidis*: PTCC1114, *Bacillus subtilis*: PTCC1023) were chosen and tested in agar disc diffusion (ADD) bioassays. The minimum inhibitory concentrations (MIC) of the active extracts were determined using nutrient broth micro-dilution (NBMD).

Bacteria were grown in nutrient broth (Merck) overnight at 37 ºC and before seeding the agar plates, their optical density were measured at 600 nm and adjusted to 0.1. An aliquot, containing 5 mg of the crude extract (dichloromethane, methanol and 80% methanol) were applied onto paper disc of 6 mm diameter. The dried papers were placed on agar seeded with 1 mL of the above bacteria suspension in a Petri dish. The Petri dishes were placed for 5 h at 4 °C that the metabolites could diffuse in the medium. The plates were incubated at 37 °C for 18 h. The antibacterial activity was determined by measuring the diameters of the clean inhibitory zone (IZ) around each paper disc. Chloramphenicol was used as the positive control (20). The most active crude extracts were found to be those that were extracted with methanol, therefore the methanol extracts of the plants (data are not shown) were chosen for MIC determination. 

*Antibacterial minimum inhibitory concentration (MIC) using nutrient broth micro-dilution (NBMD)*

NBMD was performed using serial two-fold dilution of the plant extracts added to bacterial suspension in nutrient broth as previously described (21). The plant extracts or positive control was dissolved in DMSO in different concentrations and was added (5 μL) to 95 μL of fresh media and 100 μL of bacterial suspension (OD=0.1 at 600 nm) in a 96-well microplate. The microplates were incubated at 37 ºC for 24 h in a shaking incubator and then 10 μL of 0.5% INT solution in water was added to each well and incubated for further 30 min at the above condition. The MIC was considered as the lowest concentration of the extract or antibacterial standard which discolored the purple color of the INT solution.

*Statistical analysis*

Values shown in tables 2, 3 and 4 are the average of 3-5 measurements ± SE. Correlation coefficient (R) between the two variables in table 3 were calculated by MS Excel software. The IC_50_s were calculated using Curve Expert statistical program. One-way analyses of variance (ANOVA) post hoc multiple comparison (Tukey) tests was used for the determination of signification between different measurements using SPSS software and expressed as probability factor, p-value. P ≤ 0.05 was considered to be significant. 

## Results and Discussion

*Cytotoxic activity*

Cytotoxicity of the plant extracts was measured on 3 human cancer cell lines (Table 2). 

**Table 2:** Cytotoxic activity of different extracts of *Salvia *species on human cancer cell lines

***Plant ***a	**Extraction solvent**	**IC**_50_ ** (μg/mL) **b		
		**HL60 Cells**	**K562 cells**	**MCF-7 Cells**
*Salvia aegyptiaca*	Dichloromethane	99.7 ± 3.9	97.0 ± 6.2	116.1 ± 5.0
	Methanol	NA c	NA	NA
	Methanol 80%	NA	NA	NA
*Salvia aethiopis*	Dichloromethane	44.6 ± 8.2	41.3 ± 4.5	44.4 ± 5.0
	Methanol	NA	50.1 ± 4.2	79.4 ± 12.3
	Methanol 80%	NA	NA	NA
*Salvia eremophila*	Dichloromethane	10.5 ± 0.6	15.8 ± 2.9 e	45.6 ± 1.8
	Methanol	11.9 ± 1.8	15.6 ± 2.9	47.7 ± 1.9
	Methanol 80%	24.7 ± 1.9	42.7 ± 2.5	75.2 ± 6.6
*Salvia hypoleuca*	Dichloromethane	53.3 ± 2.5	48.6 ± 2.7	83.0 ± 14.2
	Methanol	95.7 ± 6.9	93.7 ± 7.7	105.7 ± 2.1
	Methanol 80%	NA	NA	99.4 ± 10.0
*Salvia limbata*	Dichloromethane	51.0 ± 1.5	45.9 ± 0.7	64.3 ± 14.1
	Methanol	NA	111.3 ± 11.4	148.9 ± 11.8
	Methanol 80%	NA	110.6 ± 16.3	NA
*Salvia nemorosa*	Dichloromethane	NA	NA	NA
	Methanol	NA	NA	NA
	Methanol 80%	NA	87.0 ± 6.7	NA
*Salvia santolinifolia*	Dichloromethane	48.2 ± 1.4	78.7 ± 15.0	147.1 ± 68.1
	Methanol	47.0 ± 3.5	49.4 ± 1.6	108.8 ± 9.2
	Methanol 80%	49.2 ± 1.7	54.6 ± 2.2	NA
*Salvia syriaca*	Dichloromethane	NA	NA	NA
	Methanol	NA	NA	NA
	Methanol 80%	NA	NA	75.4 ± 30.3
*Salvia xanthocheila*	Dichloromethane	NA	NA	NA
	Methanol	166.9 ± 49.4	72.8 ± 9.2	127.8 ± 12.5
	Methanol 80%	NA	NA	NA
*Cisplatin *d		0.8 ± 0.1	2.1 ± 0.2	6.9 ± 1.8

a) *Salvia atropatana *and *Salvia sclarea *were also tested, but none of their extracts were active on any of the cell lines. b) Values are presented as mean ± SE of 4-5 experiments. c) NA: Not active; IC_50_ more than 200 μg/mL. d) Cisplatin was tested as a reference cytotoxic compound and found to be more active than all extracts with p ≤ 0.001 except for the dichloromethane extract of *S. eremophila.*

The most active plant was *S. eremophila, *as all of its extracts were active on 3 cell lines with IC_50_ values of 10.5-75.2 μg extract/mL. The second most active plant was *S. santolinifolia, *as all of its extracts (with the exception of 80% methanol extract on MCF-7 cells) were effective on the tested cell lines with IC_50_ values of 47.0-147.1 μg extract/mL. This indicates that there must be some compounds with different polarities in all of the extracts of these two plants that were responsible for the cytotoxic activity. The methanol and dichloromethane extracts of *S. aegyptiaca, S. aethiopis*, *S. hypoleuca *and *S. limbata *also showed significant cytotoxic activity against the tested cell lines (Table 2). However, cisplatin the anticancer positive control was several folds more active with IC_50_ values of 0.8 ± 0.1, 2.1±0.2 and 6.9±1.8 μg/mL for HL60, K562 and MCF-7 respectively, than the entire of the extracts significantly (Table 2; p ≤ 0.001) except for dichloromethane extract of *S. eremophila *p = 0.57. This shows that the activity of the extract and the drug are not different from each other. 

Recently, some of the above studied plant species were subjected to cytotoxic bioassays by different authors. For instance the methanol extracts of *S. eremophila *and *S. santolinifolia *have been examined on different human cancer cell lines, however, the cytotoxic doses have been reported above 200 μg extract/mL (9). In contrast, the triterpenoids and abietane diterpenoids isolated from the aerial parts of *S. eremophila *have shown similar cytotoxic activities on 5 human cancer cell lines, which is comparable with our results (10). In another experiment, six flavonoids with reported cytotoxic activity and rosmarinic acid were isolated from *S. limbata *(22), which may be the cause of the relatively high cytotoxicity of the extracts of this plant on cancer cells examined in this paper. 

*Antioxidant activity and total phenolic content*

Since 80% methanol is recommended as extracting solvent for phenolic compounds (23), we extracted the plants with the above solvent system and measured their antioxidant activity using DPPH radical scavenging and the total phenolic content by Folin-Ciocalteu assay (Table 3). Among the tested plants, *S. nemorosa *(IC_50_ 138.43 ± 4.6 μg PM /mL DPPH p ≤ 0.001 with all of the extracts except for *S. atropatana, S. aethiopis, S. eremophila*, *S. hypoleuca, S. santolinifolia*, and *S. sclarea*; 30.36 ± 1.08 mg EG/g PM; p ≤ 0.0001 with all of the extracts)*, S. atropatana *(IC_50_ 89.47 ± 5.97 μg PM /mL DPPH p ≤ 0.001 with all of the extracts except for *S. eremophila*, *S. nemorosa *and *S. santolinifolia*; 25.70 ± 0.04 mg EG/g PM; p ≤ 0.0001 with all of the extracts)*, S. santolinifolia *(IC_50_ 117.34 ± 4.07 μg PM /mL DPPH, p ≤ 0.04 with all of the extracts except for *S. atropatana, S. eremophila *and *S. nemorosa*; 20.21 ± 0.87 mg EG/g PM p ≤ 0.0001 with all of the extracts except for *S. eremophila, S. hypoleuca *and *S. syriaca*)*, *and *S. eremophila *

**Table 3:** Total phenolic content and DPPH radical scavenging potential of the 80% methanol extracts of plants

**Plant name **	**IC50 DPPH ** ^a^	**Total phenol** ^ b^
*S. xanthocheila*	457.00± 41.62	12.49± 1.18
*S. limbata*	557.40± 12.73	12.95± 0.70
*S. aegyptiaca*	330.4± 11.06	13.83± 0.16
*S. aethiopis*	237.37± 8.05	14.13± 0.90
*S. sclarea*	190.74± 5.7	14.83± 0.80
*S. syriaca*	315.1± 5.71	18.08 ± 0.41
*S. eremophila*	114.57± 11.5	18.86± 0.98
*S. santolinifolia*	117.34± 4.07	20.21± 0.87
*S. hypoleuca*	197.23± 6.86	20.27± 0.50
*S. atropatana*	89.47± 5.97	25.70± 0.04
*S. nemorosa*	138.43± 4.6	30.36±1.08
Quercetin	1.79±0.046	-

a) DPPH IC_50_ (μg plant extracted or μg quercetin/ 1 mL 10^-4^ M DPPH), b) Total phenol (mg eq. gallic acid in 1 g dried plant).

(IC_50_ 114.57 ± 11.5 μg PM /mL DPPH, p ≤ 0.03 with all of the extracts except for *S. atropatana, S. nemorosa *and *S. santolinifolia*; 18.86 ± 0.98 mg EG/g PM, p ≤ 0.002 with all of the extracts except for *S. santolinifolia, S. hypoleuca *and *S. syriaca*) were the most active radical scavengers with the highest total phenol contents. However, *S. limbata, S. xanthocheila, S. aegyptiaca *and *S. aethiopis *in increasing order of efficiency, had the lowest radical scavenging potential (DPPH IC_50S_ 557.40 ± 12.73 to 237.37 ± 8.05 μg PM /mL DPPH) with lower total phenol contents compared to the above mentioned plants (Table 3). There was not a perfect correlation (R2 = 0.48; R = -0.69) between antioxidant and total phenol content data in our test (Table 3). This may be due to the presence of different types of phenolic compounds in the plant extracts. However the IC_50_ of quercetin, the standard natural antioxidant is 1.79 ± 0.046 μg/mL DPPH which is significantly different from all of the tested plants extracts reported here (p ≤ 0.0001). Several structure-activity-relationship studies reported the effect of the number and position of phenolic hydroxyls in the radical scavenging potential of the phenolic compounds (24); for instance when one of the *ortho*- or *para*- free hydroxyls in a phenolic compound are protected by glycosylation or methylation, then its radical scavenging activity is dramatically decreased (18). 

The results of this study are consistent with our previous findings on screening of some of the Lamiaceae plants (25). Among the extracts of twenty-four plants of the family Lamiaceae, the extracts of *Salvia *in general were the most active radical scavengers among which *S. eremophila *and *S. santolinifolia *were the most active ones (25). In another study, the essential oil and methanolic extract of *S. eremophila *were subjected to DPPH bioassay (26). Only the methanolic extract of the plant significantly reduced the reagent (26). Examination of antioxidant and total phenol contents of six *Salvia *species has resulted in determination of *S. xanthocheila *and *S. sclarea *as the weakest radical scavengers in those plant series (27). *S. hypoleuca *and four other sage plants were assessed for their DPPH antioxidant activity and total flavonoid contents, but no favorable correlation was detected between the tests results in different plants (28). Fourteen Turkish sage species were examined for their antioxidant activity using DPPH reagent, among which different extracts of *S. sclarea *and *S. syriaca *showed similar inhibition percentage; however, these two plant extracts had lowest antioxidant activity (29). On the other hand, these plants are considered as medium radical scavengers in our sage series (Table 3). 

*Antimicrobial activity of the sage extracts*

Antimicrobial activity of the methanol extracts of the plants were measured on 6 different Gram-negative and Gram-positive bacteria (Table 4). *S. eremophila, S. limbata, S. santolinifolia *and *S. sclarea *were the most active plants and inhibited the growth of all tested microorganisms at MIC values between 0.31-5 mg/mL on the tested microorganisms (Table 4). On the other hand, *S. aegyptiaca *and *S. aethiopis *were only active at MIC 5 mg/mL against the growth of *S. typhi *and therefore resulted the weakest plants in the antimicrobial bioassay. The gram positive bacteria with MICs 0.0125 for *B. subtilis *and *Staph. aureus *and 0.025 for *Staph. epidermidis *were more susceptible to chloramphenicol than the gram negative ones with MICs 0.05 mg/mL media.

Most of the studies performed on this genus in Iran evaluated the antimicrobial activity of the essential oils (3). Both essential oil and methanol extract of *S. eremophila *showed relatively strong antimicrobial activity against Gram-positive and Gram-negative bacteria including *E. coli*, *B. subtilis*, *Staph. aurous *and *Staph. epidermis *(26). These findings are similar to MIC values that we obtained for the methanolic extract of this plant on the same bacteria (Table 4). The antimicrobial and antioxidant activity of methanolic extract of *S. aegyptiaca *from Tunisia were evaluated and found to be the most active one among other *Salvia *species tested (30).

**Table 4:** Antimicrobial potential (MICa) of different plant extracts by nutrient-broth micro-dilution bioassay

***Plant name***	***E. coli *** ***a***	***K. pneumoniae***	***S. typhi***	***B. subtilis***	***Staph. epidermidis***	***Staph. aureus***
*S. aegyptiaca*	NA b	NA	5	NA	NA	NA
*S. aethiopis*	NA	10	5	NA	NA	NA
*S. atropatana*	2.5	NA	5	2.5	NA	NA
*S. eremophila*	2.5	2.5	0.31	2.5	0.3	0.6
*S. hypoleuca*	NA	2.5	5	NA	1.25	1.25
*S. limbata *	5	2.5	1.25	5	2.5	2.5
*S. nemorosa*	NA	NA	10	NA	NA	NA
*S. santolinifolia*	2.5	5	1.25	2.5	1.25	2.5
*S. sclarea*	5	2.5	0.31	5	0.31	1.25
*S. syriaca*	NA	NA	2.5	NA	2.5	1.25
*S. xanthocheila*	5	2.5	2.5	2.5	NA	1.25
*Chloramphenicol *	0.05	0.05	0.05	0.0125	0.025	0.0125

a) Minimum inhibitory concentration (MIC) of the plant extracts in the bacterial suspension in the nutrient broth media (mg/ mL). b) NA= not active.

## Conclusion

The plants of the genus *Salvia *are rich in antioxidant polyphenols (7, 31, 32) and abietane diterpenoids such as rosmarinic acid (7, 31, 33) and carnosol and carnosic acid (34). The diterpenoids isolated from shoots and roots of different *Salvia *species showed considerable anticancer activity (35, 36) as well as antimicrobial (1, 5, 8, 37) properties. Therefore we choose the above plants for screening their extracts for the above mentioned bioassays. Different cytotoxic, antioxidant and antimicrobial potential of various extracts of the sage plants indicate that we can use these data to choose the appropriate extract of the plants for further purification and identification of their active ingredients. *S. eremophila *and *S. santolinifolia *are the two most interesting bioactive plants in this study and we selected them for further investigation of their active constituents. The aqueous methanolic extracts of *S. nemorosa *and *S. atropatana *are suggested for determination of their antioxidant constituents in the current paper. *S. limbata*, *S. xanthocheila *and *S. sclarea *are suggested to be analyzed for their antimicrobial constituents. Finally, since *S. hypoleuca *and *S. syriaca *are two plants with very rare sesterterpenes, they may also be good candidates for evaluation of their terpenoids in the cytotoxic bioassays.
